# Buckling Analysis of Thin-Walled Composite Structures with Rectangular Cross-Sections under Compressive Load

**DOI:** 10.3390/ma16216835

**Published:** 2023-10-24

**Authors:** Patryk Rozylo, Michal Rogala, Jakub Pasnik

**Affiliations:** Department of Machine Design and Mechatronics, Faculty of Mechanical Engineering, Lublin University of Technology, Nadbystrzycka 36, 20-618 Lublin, Poland; m.rogala@pollub.pl (M.R.); j.pasnik@pollub.pl (J.P.)

**Keywords:** buckling, closed composite profiles, experimental studies, numerical simulations, axial compression

## Abstract

The purpose of this research was the analysis of the stability of compressed thin-walled composite columns with closed rectangular cross-sections, subjected to axial load. The test specimens (made of carbon–epoxy composite) were characterized by different lay-ups of the composite material. Experimental tests were carried out using a universal testing machine and other interdisciplinary testing techniques, such as an optical strain measurement system. Simultaneously with the experimental studies, numerical simulations were carried out using the finite element method. In the case of FEA simulations, original numerical models were derived. In the case of both experimental research and FEM simulations, an in-depth investigation of buckling states was carried out. The measurable effect of the research was to determine both the influence of the cross-sectional shape and the lay-up of the composite layers on the stability of the structure. The novelty of the present paper is the use of interdisciplinary research techniques in order to determine the critical state of compressed thin-walled composite structures with closed sections. An additional novelty is the object of study itself—that is, thin-walled composite columns with closed sections.

## 1. Introduction

Thin-walled composite materials—carbon-epoxy laminates—are a special group of structures that are used in the aerospace, automotive, or construction industries. Most often, these thin composite materials are made using carbon fiber–epoxy resin (CFRP) [1,2] or glass fiber–epoxy resin (GFRP) [3,4] configurations and are characterized by both open [5,6] and closed cross sections [7,8,9,10,11]. The above-mentioned composite materials are characterized by a certain behavior that occurs due to compression load [12,13]. The issue is commonly known as loss of stability (buckling) [14,15] associated with the accompanying deformation of the column. It is possible to distinguish several stages of compression of thin-walled columns made of composites. Initially, the walls of the construction are only compressed (pre-buckling stage), after which buckling occurs due to further loading (buckling stage), and then, when the equilibrium path is stable, increasing loading is accompanied by an increase in deflection (post-buckling stage) [16]. The issue of loss of stability has been addressed in many scientific papers and is still relevant due to the possibility of modifying the properties of the composite material [17,18,19].

Analysis of the critical state shows that the values of the failure load can even be several times higher than the critical load [20,21,22]. The correct orientation of the fibers and the number of layers can provide the thin-walled composite materials with a different range of stiffness, which translates into the behavior characteristics of the construction [23,24,25]. Accurate analysis of the critical state allows us to determine the form of buckling and the corresponding value of the critical load. In experimental studies, the value of critical load is determined based on approximation methods presented in many scientific papers [26,27]. These methods involve estimating the value of the critical load on the basis of experimental equilibrium post-buckling paths. A detailed description of the methods for determining the approximate value of the critical load is presented in many scientific papers, where a group of results and the methods of analysis are presented [28]. For numerical simulations, the critical load and the form of buckling are determined from the linear eigenproblem solution [27].

The aim of analyzing the process of axial compression of composite columns with closed cross-sections requires the use of several independent test methods. The evaluation of the behavior of the structure in the case of experimental testing was based on a universal testing machine, an acoustic emission testing system, and an optical system for measuring the deformation of thin-walled composite materials [29,30]. Coupled tests based on several independent methods make it possible to determine the limit states of the construction, the deformations obtained, and the values of critical forces [31,32]. The current paper contains a comparative stability analysis of two types of columns with closed cross-sections.

The novelty of the present research mainly includes:

- The use of interdisciplinary testing methods for structural stability assessment (testing machine, optical deformation measurement system, numerical FEA simulations);
- Manufacture of a new object of research in the form of thin-walled carbon-epoxy composite materials with closed sections;
- Study of the influence of the lay-up of the composite layers and the shape of the cross-section of the composite materials on the critical state.

The manufactured thin-walled composite materials with closed sections made of CFRP composite were developed through a project from the National Science Centre (Poland)—project number 2021/41/B/ST8/00148.

## 2. Subject of the Study

**Figure 1:**
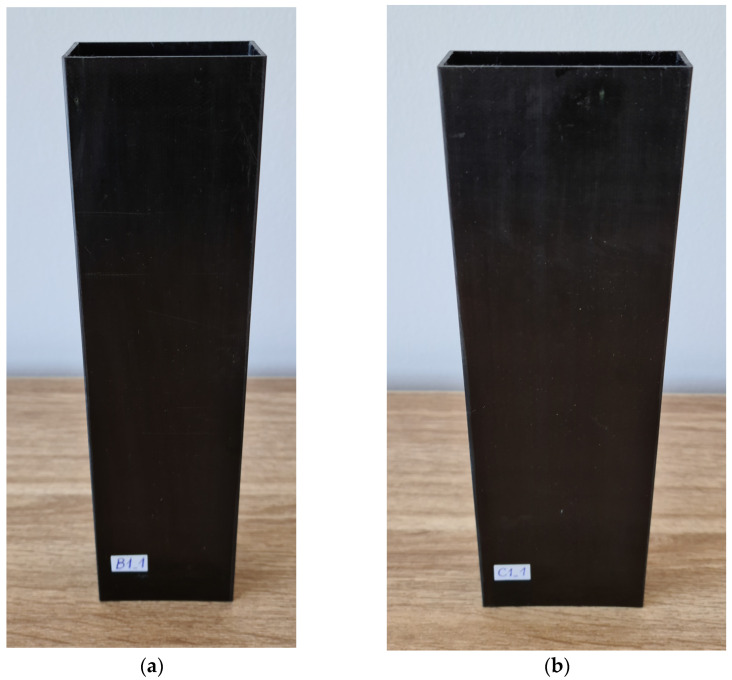
Experimental specimens: (**a**) B—column, (**b**) C—column.

The study focused on thin-walled composite profiles made of carbon fiber-reinforced polymer (CFRP). Each profile consisted of eight layers of CFRP [33]. This paper describes two different types of profiles, denoted as B and C, with the following dimensions: 30 mm × 50 mm and 20 mm × 60 mm, respectively, with a wall thickness of 1.2 mm. The profiles had a maximum height of 200 mm. The following stacking sequences were utilized: B1/C1—[0°/45°/−45°/90°]s, B2/C2—[0°/90°/0°/90°]s, B3/C3—[45°/−45°/90°/0°]s, B4/C4—[90°/−45°/45°/0°]s. The sequences of layer configurations were derived from preliminary numerical simulations (which made it possible to predetermine critical loads and the form of buckling in order to preserve variety in the study of construction stability). For each of the layup configurations, three specimens were made. Note that every layout was symmetrical with respect to the center surface, as indicated by the subscripts next to the layout of the layer sequence. The columns were manufactured with autoclave technology using prepreg tapes with the trade name: CYCOM 985-42%-HS-135-305 (Solvay, Tempe, AZ, USA). For the production of the prepreg, epoxy resin type 985 was used, while the reinforcement was high-strength (HS) carbon fibers with a density of 135 g/m^2^. The volume fraction of the resin in the prefabricated material was 42%. Profiles were made by winding a 305 mm wide prepreg tape at the desired angle, corresponding to the sequence of layers in the final product, on a properly prepared inner core. The parameters of the autoclaving curing process were set at a temperature of 177 °C and a pressure of 0.6 MPa and monitored throughout the course of the process. The production of the profiles was carried out by an external company specializing in making composite parts using an autoclave technique. The expertise of the contractor resulted in top-quality profiles with high repeatability of mechanical properties and dimensions. The quality of the profile fabrication was checked by using several techniques, including the use of the Keyence VHX 970F digital microscope (Keyence, Mechelen, Belgium) [34]. This microscope, equipped with a dedicated mobile head, allowed thorough observation of the structure and digital image capture. Figure 1 shows examples of ready-made profiles for experimental studies.

To obtain the material properties of the CFRP, test specimens for the determination of material data were made in accordance with the ISO standards [35]. Static tensile tests were carried out under the requirements and restrictions outlined in PN-EN ISO 527-5 (of 2010) [36] of which ASTM D 3039 [37] was the equivalent. Subsequent tests were performed as static shear tests based on PN-EN ISO 14129 (of 2000) [38]—the equivalent of ASTM D 3518 [39]. Finally, static compression tests were performed in accordance with PN-EN ISO 14126 (of 2002) [40]; the American Standard equivalent was ASTM D 3410 [41]. The process of manufacturing the specimens, their preparation for testing, and the tests themselves are described in detail in the paper [42]. The above-mentioned paper presents the methodology for determining the required material parameters of CFRP extensively. The data derived from these tests are shown in Table 1 [42,43].

## 3. Experimental Study

Interdisciplinary research methods were used to perform the experimental tests. Experimental studies were conducted in order to determine the stability of composite materials [42]. All the above-mentioned tests were conducted on a Zwick Z100 universal testing machine (ZwickRoell GmbH & Co. KG, Ulm, Germany) [22,29]. The next stage of the research was to run axial compression tests on thin-walled composite structures at room temperature. The crosshead of the testing machine was moving at a rate of 1 mm/min. The effect of the tests was to obtain the critical state by observing the formation of the buckling of the profile and the subsequent determination of the critical load using approximate methods [16,28]. To determine the critical force, one of the approximation methods was chosen—the method of intersection of straight lines [26]. To determine the approximate value of the critical load using this method, a load-displacement or, in other words, a load-shortening curve for the chosen structure was required. The chosen method involves approximating with a linear function two appropriately selected areas of the experimental curve, one before the point of change in “stiffness” within the force-displacement curve and the other after the change in “stiffness”. The selected areas cannot be arbitrary; the requirement for the correct determination of the critical force by the method of intersection of straight lines is the selection of the areas of the force-displacement characteristics that are most nearly aligned with the straight line. Making the convergence between the two lines as high as possible means keeping the correlation coefficient *R*^2^ as close as possible to the value of 1. In practice, the value of the coefficient *R*^2^ cannot decrease below 0.95. The closer to the value of 1 one is, the better the obtained results will be. Ideally, this coefficient is 1. In order to correctly determine the critical force, the matrix method (determinant method) was used.

As basic geometric relationships indicate, two lines that are not parallel to each other intersect at a certain point. The point of intersection is located on both lines at the same time, so the coordinates must concurrently satisfy the equations of both lines. These coordinates can be obtained by solving a simple system of two linear equations:

(1) $$\{\begin{array}{c} A_{1}x+B_{1}y+C_{1}=0\\ A_{2}x+B_{2}y+C_{2}=0 \end{array}$$

where *A*_1_ and *A*_2_ are the values of the directional coordinates of the lines at *x*, *B*_1_ and *B*_2_ are the values of the coefficients at *y*, while *C*_1_ and *C*_2_ are the numerical values that determine the so-called free expression of the function.

For determining the intersection point, Equation (1) must be rearranged to the form depicted in Equation (2):

(2) $$\{\begin{array}{c} A_{1}x+B_{1}y=-C_{1}\\ A_{2}x+B_{2}y=-C_{2} \end{array}$$

The system of first-degree equations in the form shown in Equation (2) with two unknowns may be solved employing the method of determinants of matrices as follows:

(3) $$W=\left|\begin{array}{cc} A_{1} & B_{1}\\ A_{2} & B_{2} \end{array}\right|=A_{1}\cdot B_{2}-A_{2}\cdot B_{1}$$

(4) $$W_{x}=\left|\begin{array}{cc} -C_{1} & B_{1}\\ -C_{2} & B_{2} \end{array}\right|=\left(-C_{1}\right)\cdot B_{2}-\left(-C_{2}\right)\cdot B_{1}$$

(5) $$W_{y}=\left|\begin{array}{cc} A_{1} & -C_{1}\\ A_{2} & -C_{2} \end{array}\right|=A_{1}\cdot \left(-C_{2}\right)-A_{2}\cdot \left(-C_{1}\right)$$

Under the initial assumptions that the above-mentioned lines are nonparallel, and for *W* ≠ 0, the system of equations is marked and has exactly a single solution:

(6) $$\{\begin{array}{c} x=\frac{W_{x}}{W}\\ y=\frac{W_{y}}{W} \end{array}$$

where *x* and *y* are the coordinates of the intersection point of two straight lines.

Consequently, the approximation method made it possible to determine the approximate value of the critical load within the experimental load-shortening curve. 

**Figure 2:**
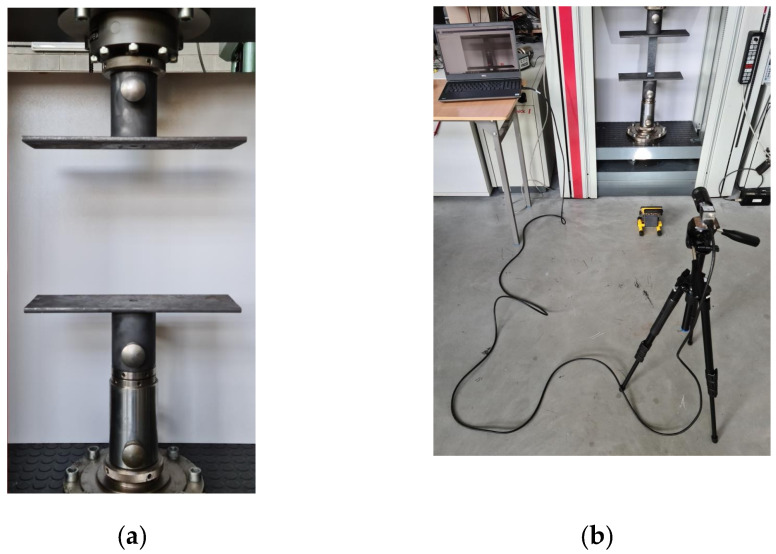
Experimental test stand: (**a**) experimental test heads, (**b**) general view of the test stand—Zwick Z100 testing machine with Aramis 2D system.

Moreover, experimental studies also allow one to determine the path of post-buckling equilibrium. Such studies are carried out until the complete failure of the specimen and provide an opportunity to capture the ultimate failure force, i.e., the maximum load that the profile can carry. These tests were conducted on a universal testing machine, as mentioned elsewhere. The total number of specimens tested was 24 (12 specimens of type B and 12 specimens of type C). Axial compression tests were performed using special heads with flat working surfaces that were parallel to each other. These heads were rigidly attached to the bottom of the testing machine and to the top crosshead. Figure 2a illustrates the test stand with the heads installed on the machine. In addition, a vision-based system for measuring the deformation of the profile at the very moment of critical load application—the ARAMIS 2D digital image correlation system [44,45]—was used. The use of the referred device enables, in particular, the observation and measuring of deformations at the moment of the loss of stability of the structure (buckling). Figure 2b presents the test stand with the vision system employed. In order to obtain valid deformation values using the ARAMIS 2D system, dedicated non-reflective, red-colored mats were used as a background for the tests. When too much illumination is applied to the specimen during the test, unwanted overexposed areas appear within the composite profile, which have an adverse effect on the deformation registration of the structure. The use of a non-reflective background eliminated the problem with overexposed areas due to the fact that the mats absorb excess illumination and neutralize this unwanted effect. In order to obtain accurately captured images of profiles in the axial compression test, proper lighting is required, which was achieved using LED lamps.

In addition, the AMSY-5 acoustic emission measurement system was also used in the experimental studies. By recording signals such as number of counts, number of hits, amplitude and energy, the state of the structure and its damage could be assessed. 

Experimental studies made it possible to determine both the values of critical loads and the structure’s buckling forms. The former was determined by means of the method of the intersection of straight lines while the latter was established through the structure’s deformations obtained using a digital image correlation system during the tests. 

## 4. Numerical Simulations

Numerical studies were based on the finite element method and were conducted using Abaqus software (Abaqus 2023, Dassault Systemes Simulia Corporation, Velizy Villacoublay, France). The numerical studies used a Lamin-type material model, the data of which was described in more detail during the presentation of the research subject. All numerical studies were carried out in two steps. The first stage was determining the linear stability of the structure (buckling) within the framework of which the linear eigenproblem was solved, based on the criterion of minimum potential energy. In view of the above, the buckling form of the thin-walled composite column was determined, along with the determination of the value of the critical load, corresponding to the obtained buckling form. The value of the critical load was determined by defining the unit load of the structure, which made it possible to determine the critical state [29]. The following is the relationship that allows the calculation of the critical load (7), it comes directly from the documentation of the FEM software (Abaqus 2023):

(7) $$\left(K_{0}^{\mathrm{NM}}+\lambda_{i}K_{\Delta }^{\mathrm{NM}}\right)v_{i}^{M}=0$$

where $K_{0}^{\mathrm{NM}}$ is structural stiffness matrix relating to the baseline (includes preload effects *P*^N^), $K_{\Delta }^{\mathrm{NM}}$ refers to the differential matrix of initial stress and load stiffness caused by the incremental loading pattern (*Q*^N^), *λ_i_* illustrates the eigenvalues, *v*^M^ is the buckling mode (known as the eigenvectors), ^M^ and ^N^ refer to degrees of freedom M and N of the whole model, and *i* refers to the _I_ th buckling mode. Furthermore, the critical buckling loads represent then *P*^N^ + *λ_i_Q*^N^. Additionally, *v*^M^ is normalized vectors (do not reflect the actual quantities of strain at critical load). They are normalized so that the maximum component of displacement is 1.0. When all components of displacement are zero, the maximum component of rotation is normalized to 1.0. Once damage is initiated, further loading of the composite structure will degrade the stiffness parameters of the material.

**Figure 3:**
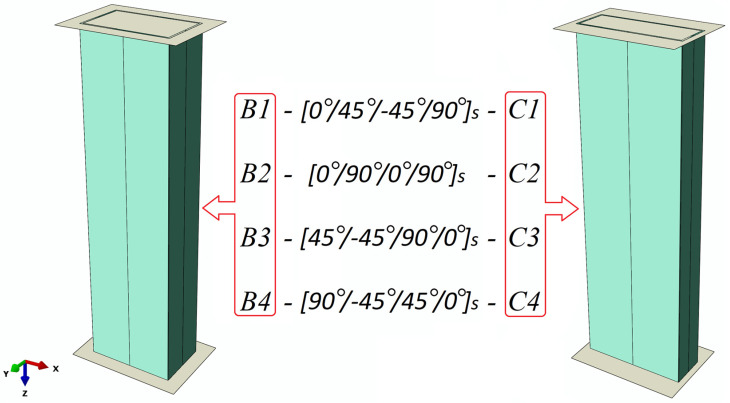
Numerical model with listed configurations of composite material layers for two types of columns.

The numerical model consisted of a composite structure and non-deformable plate elements, which allowed correct modelling of the boundary conditions. The composite column with rectangular cross-section consisted of eight layers of composite material (CFRP) of equal thickness for both B- and C-type specimens. The numerical model included four different arrangements of fiber composite orientation shown in Figure 3. The composite structure had the same geometric parameters regardless of the arrangement of the composite material layers used. Both experimental studies and numerical simulations considered the following cases of arrangement of composite material layers: *B*1 and *C*1—[0°/45°/−45°/90°]s, *B*2 and *C*2—[0°/90°/0°/90°]s, *B*3 and *C*3—[45°/−45°/90°/0°]s, *B*4 and *C*4—[90°/−45°/45°/0°]s, as shown in Figure 3.

**Figure 4:**
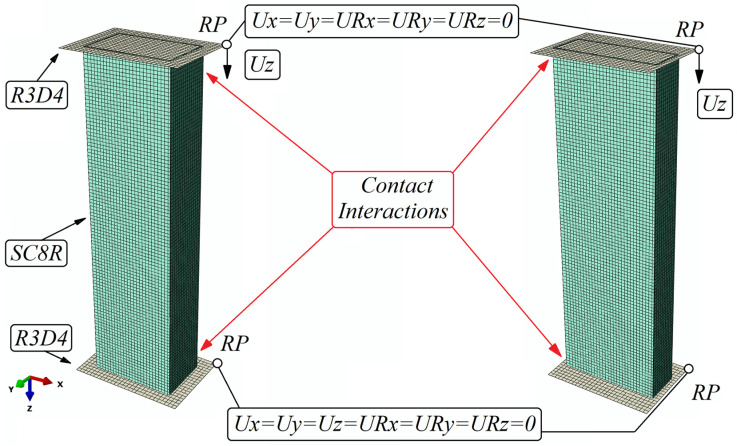
Discrete model with defined boundary conditions.

The discrete model was formulated using Continuum Shell elements (with a physical representation of the thickness of the composite material, which included eight layers of composite material), whereas the plate elements serving as supports were modelled using Shell elements. The composite structure consisted of SC8R-type finite elements (8-node quadrilateral continuous general-purpose shell in-plane, reduced integration with hourglass control, finite membrane deformations, having three translational degrees of freedom per computational node). In contrast, the supports were defined by non-deformable finite elements of type R3D4 (4-node three-dimensional rigid quadrilateral, having six degrees of freedom (three translational and three rotational) per computational node). A mesh density of 2 mm was used for the composite structure, while 2.5 mm was used for the non-deformable plates. The discrete model consisted of 10,320 finite elements (9200 linear hexahedral elements of type SC8R and 1120 linear tetrahedral elements of type R3D4). Contact properties representing the interaction of the contacting surfaces were reflected by using normal and tangential contact (friction coefficient 0.2). To represent the correct behaviors of the structure, boundary conditions were applied by assigning the load to reference points assigned to the lower and upper non-deformable plate, respectively. The upper plate, acting as the loading element, had all degrees of freedom locked, with the exception of the displacement relative to the Z axis, on which the load was applied. The bottom plate serving as the base had all rotational as well as translational degrees of freedom locked. The load was realized with a displacement relative to the Z axis. A discrete model of the structure with defined boundary conditions is shown in Figure 4. The numerical model presented below was used to perform a simulation using the finite element method of stability (buckling) of thin-walled structures.

## 5. Research Results

In the course of the experimental research and numerical simulations using the finite element method, it was possible to assess the stability of thin-walled composite structures, which is important for the evaluation of composite structures for the use of such components in the aerospace or automotive industries. Experimental research used interdisciplinary testing techniques to assess the structural stability, while in the case of numerical simulations, it was possible to determine critical (buckling) states using an advanced model of the composite material.

The main purpose of the research conducted was to analyze the critical state. The research included both an experiment on physical specimens and a numerical study using the finite element method. The analysis of the critical state for physical specimens was carried out using a universal testing machine, where the occurring form of buckling was observed in axial compression of the structure using an optical strain measurement system, while the critical load values were determined based on the approximation method of intersecting straight lines. The method of determining the critical load for the described method is presented in Equations (1)–(6) in Section 3. The procedure for estimating the critical load values for all experimentally tested specimens was the same. To determine the critical load value, we relied on load-displacement curves obtained from bench tests. The effective approximation ranges for the experimental curves (the range before and after the change in the “stiffness” of the experimental curve) were approximated by using linear functions while maintaining the correct correlation coefficient between the approximation functions and the selected approximation ranges at the highest possible level of *R*^2^ ≥ 0.95. All tested cases obtained a coefficient value that was significantly higher, oscillating above *R*^2^ ≥ 0.99, which indicates the high accuracy of the realized tests. Therefore, linear approximating functions were determined, which allowed further calculation of approximate values of critical forces. The value was determined by solving a system of equations, that is, determining the point of intersection of the approximating functions. As an example of the first sample B1_1, the methodology for determining the critical load approximation is presented, in which two approximation functions are initially compared using a system of equations:

(8) $$\{\begin{array}{c} A_{1}x+B_{1}y+C_{1}=24,008.79x-1y-1622.46=0\\ A_{2}x+B_{2}y+C_{2}=16,798.83x-1y+4850.59=0 \end{array}$$

To determine the point of intersection, the notation resulting from Equation (8) must be transformed to another form, consistent with the following notation (9):

(9) $$\{\begin{array}{c} A_{1}x+B_{1}y=-C_{1}\;↔\;24,008.79x-1y=1622.46\\ A_{2}x+B_{2}y=-C_{2}\;↔\;16,798.83x-1y=-4850.59 \end{array}$$

The obtained system of first-degree equations with two unknowns is solvable by the matrix determinant method (10)–(12):

(10) $$W=\left[\begin{array}{cc} A_{1} & B_{1}\\ A_{2} & B_{2} \end{array}\right]↔\left[\begin{array}{cc} 24,008.79 & -1\\ 16,798.83 & -1 \end{array}\right]=-7209.96$$

(11) $$W_{x}=\left[\begin{array}{cc} -C_{1} & B_{1}\\ -C_{2} & B_{2} \end{array}\right]↔\left[\begin{array}{cc} 1622.46 & -1\\ -4850.59 & -1 \end{array}\right]=-6473.05$$

(12) $$W_{y}=\left[\begin{array}{cc} A_{1} & -C_{1}\\ A_{2} & -C_{2} \end{array}\right]↔\left[\begin{array}{cc} 24,008.79 & 1622.46\\ 16,798.83 & -4850.59 \end{array}\right]=-143,712,226.41$$

With the initial assumption that the aforementioned lines are not parallel, with *W* ≠ 0, the system of equations is determined and has exactly one solution (13):

(13) $$\{\begin{array}{c} x=\frac{W_{x}}{W}=0.90\\ y=\frac{W_{y}}{W}=19,932.46 \end{array}$$

**Figure 5:**
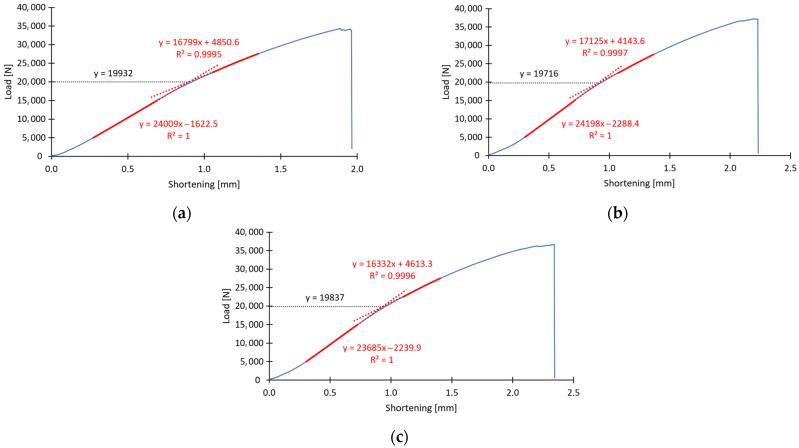
Experimentally determined critical load: (**a**) specimen B1_1, (**b**) specimen B1_2, (**c**) specimen B1_3.

**Figure 6:**
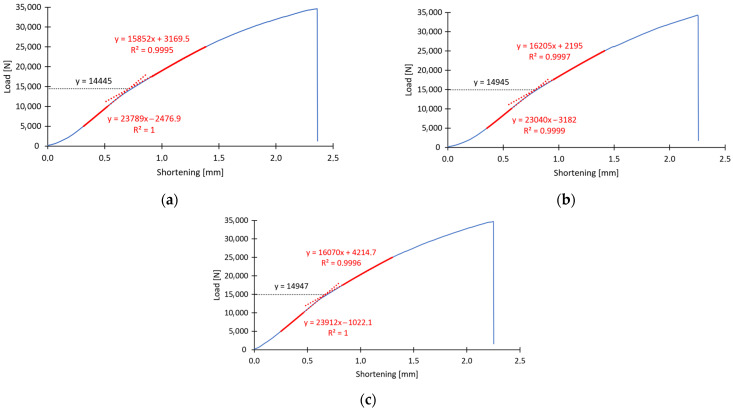
Experimentally determined critical load: (**a**) specimen C1_1, (**b**) specimen C1_2, (**c**) specimen C1_3.

With the method described above, the approximate critical load value was determined for the first specimen of type B, designated B1_1. Thus, it was determined that the critical load value, causing loss of stability of the thin-walled composite structure, is approximately *P*_cr_ = 19,932 N and occurs when the structure is shortened by *u* = 0.90 mm (vertical displacement of the crosshead of the testing machine). The above-described method was used to derive the critical load values for all specimens in the experimental tests. Figure 5 and Figure 6 show graphically how the critical load was determined for the six selected specimens, i.e., *B*_1_ and *C*_1_ (three specimens of each column type).

In Figure 5 and Figure 6, the depicted lines indicate successively: red dashed line—approximation function, blue solid line—experimental curve, red solid line—effective range of approximation, black dashed line—line representing critical load. The determined values of critical forces made it possible to compare the tested specimens in terms of the influence of the arrangement of the fiber composite layers on the stability of the structure. In order to better present the obtained experimental results, the values were presented in Table 2 and Table 3 for specimen types B and C, respectively.

It was determined that the highest critical load values were obtained by the B3 and C3 type profiles—characterized by composite material layer arrangements [45°/−45°/90°/0°]s, where the average critical load value was *P*_cr_ = 21,947 N for the B3 model and *P*_cr_ = 17,201 N for the C3 model. The composite columns with the lowest critical load were characterized by B4 [90°/−45°/45°/0°]s and C2 [0°/90°/0°/90°]s, where the average load values were *P*_cr_ = 17,108 N and *P*_cr_ = 13,485 N, respectively. In describing the type C column, it is worth noting that models C2 and C4 had very similar values of critical loads. In the case of specimens C2_3 and C4_3, it was the C4 column that obtained a lower value of critical load, according to Table 3. Based on the results of the average values of critical load, it was determined that specimens of type B3 showed about 1.28 times higher load than specimens of type B4, in the case of model C it was 1.28 for specimens of type C3 and C2, respectively. Analyzing the extreme results, i.e., the highest value of critical load (sample B3_3) and the lowest value of critical load (sample B4_3), it was determined that the ratio of maximum to minimum load was 1.33. A similar comparison of extreme values for column type C showed a ratio of load values of 1.38 between samples C3_2 (*P*_cr_ = 18,041 N) and C4_3 (*P*_cr_ = 13,091 N). 

It was also noted that buckling of the structure occurs at different deflection values, i.e., in the case of type B3 profiles, it occurs when the structure is shortened by *u* = 0.95 mm, while in the case of type B4 profiles, it occurs when the structure is shortened by *u* = 0.81, which is about a 0.14 mm difference between the above-mentioned structure types. In the case of the type C column, the extremes of deflection at which the loss of stability occurred were *u* = 0.91 mm (C3) and *u* = 0.55 (C2) on average. Thus, it was concluded that the arrangement of fiber composite layers has a major impact on the stability of thin-walled composite structures with a closed square section. In addition, it is noticeable that there are significant differences in the values of critical loads and deflections at which stability is lost for the two types of columns analyzed (B and C). The thin-walled column with a cross-section of 20 × 60 mm (type C) was characterized by a lower critical load. The described effect is observed when comparing all layer arrangements (1–4) shown for columns B and C of Table 2 and Table 3. 

**Figure 7:**
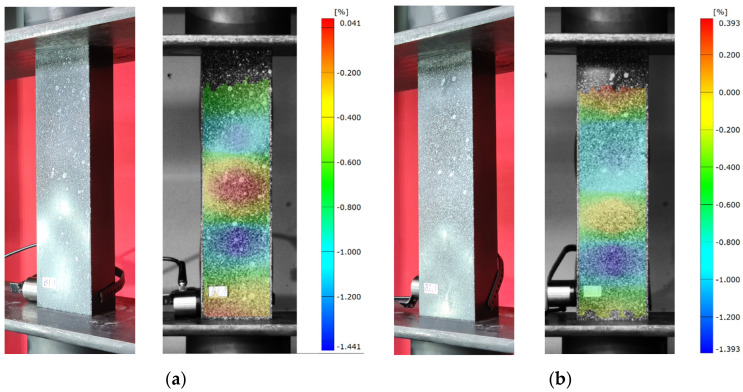

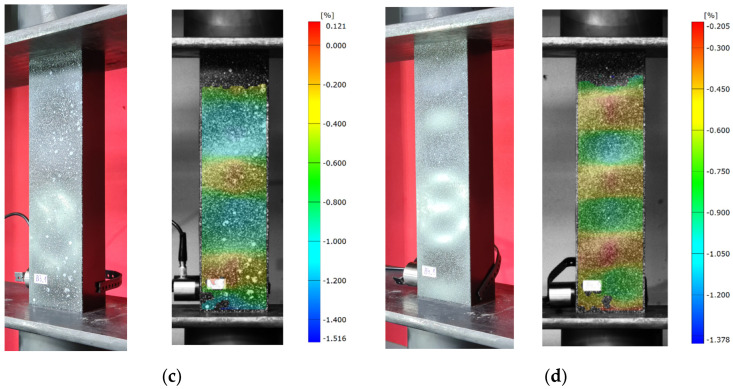
Loss of structural stability—experimental studies: (**a**) specimen type B1, (**b**) specimen type B2, (**c**) specimen type B3, (**d**) specimen type B4.

**Figure 8:**
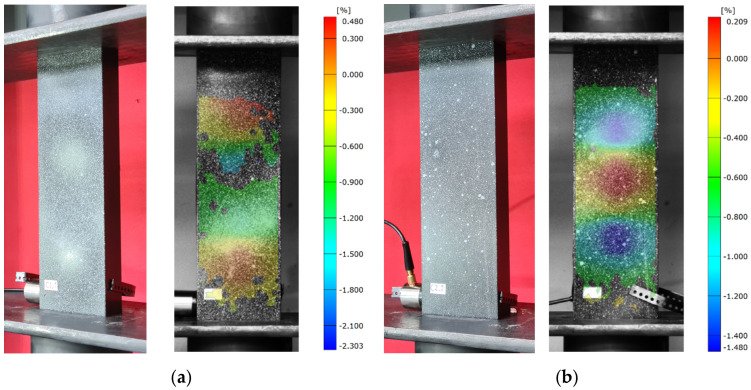

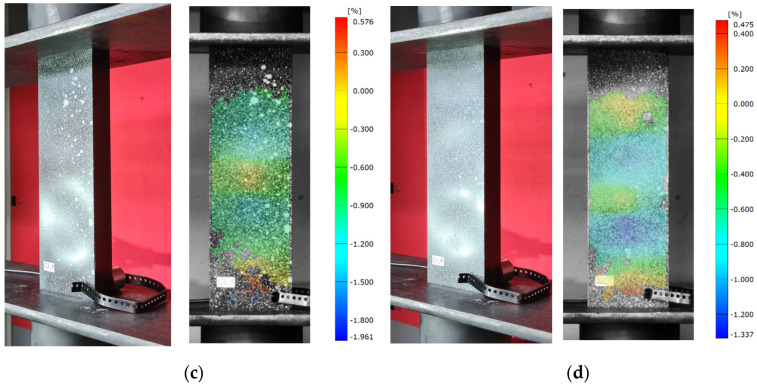
Loss of structural stability—experimental studies: (**a**) specimen type C1, (**b**) specimen type C2, (**c**) specimen type C3, (**d**) specimen type C4.

In addition, a qualitative evaluation of the critical condition was carried out in the experimental study. The study consisted of recording buckling forms obtained by capturing images of each type of composite profile during loss of stability (buckling), as well as recording buckling forms using an optical strain measurement system—Aramis 2D. In the case of the Aramis 2D optical system, it was necessary to use special filters applied directly in the software, highlighting the buckling form (registration of deformations in the longitudinal direction of the structure with a median filter). The registered experimental buckling forms are shown below (Figure 7 and Figure 8).

During the execution of the experimental tests, it was observed that for the tested profiles there were specific numbers of half-waves in the longitudinal direction of the column: B1—three half-waves, B2—four half-waves, B3—five half-waves, and B4—seven half-waves. In the case of the C-type model, a different number of half-waves was observed for specific layer arrangements, whereas the values obtained reflected the results obtained with numerical simulations using FEM. 

**Figure 9:**
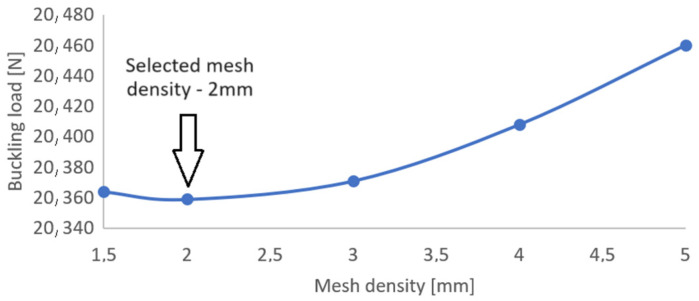
The influence of mesh density on the buckling load value (on the specimen B1).

For numerical simulations using FEM, the critical state analysis was carried out based on the solution of a linear eigenproblem. During the preparation of numerical models, the effect of mesh density on the value of critical load was made (Figure 9). The study was carried out on a sample specimen B1 that made it possible to estimate the value of critical load—the most consistent with experimental results (a mesh density of 2 mm was adopted).

**Figure 10:**
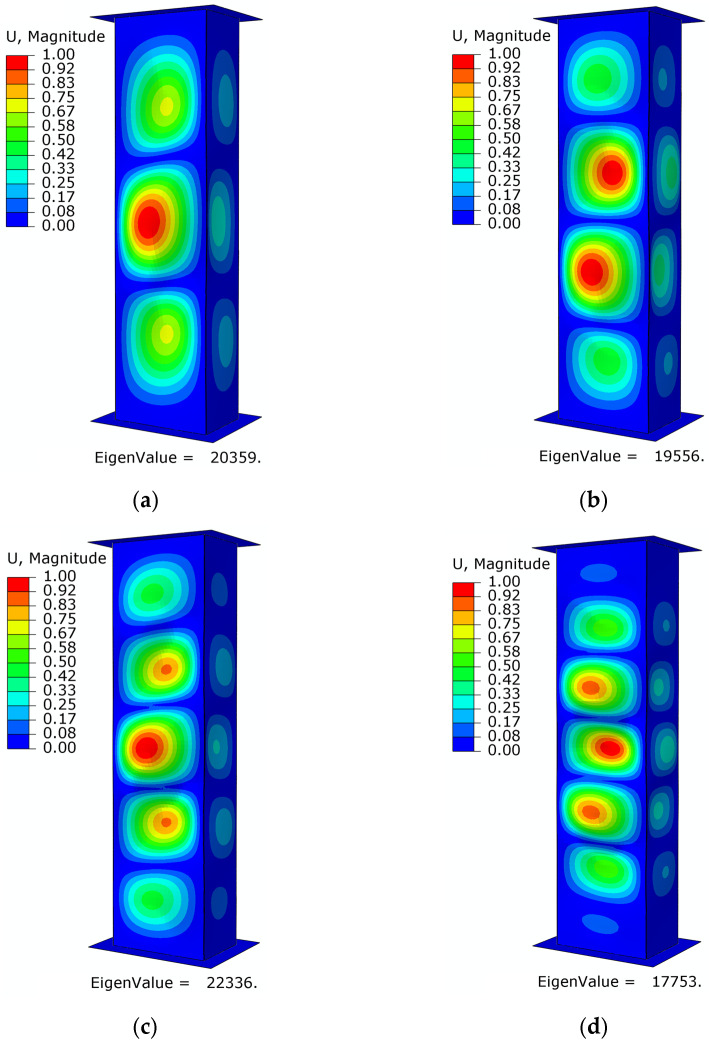
Loss of structural stability—numerical studies: (**a**) specimen type B1, (**b**) specimen type B2, (**c**) specimen type B3, (**d**) specimen type B4.

**Figure 11:**
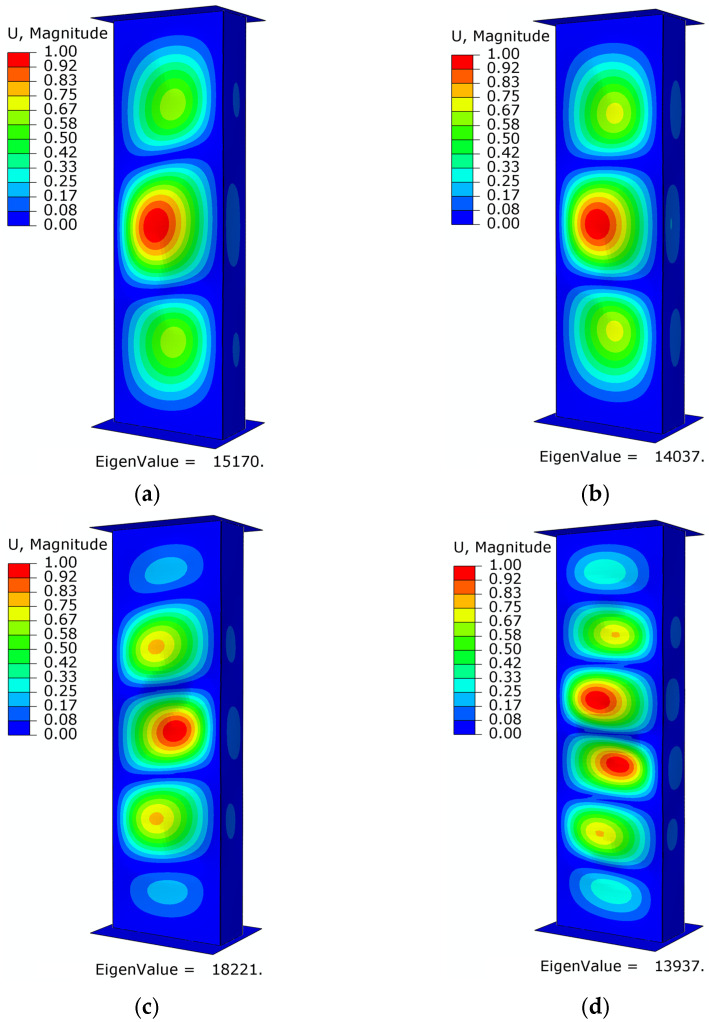
Loss of structural stability—numerical studies: (**a**) specimen type C1, (**b**) specimen type C2, (**c**) specimen type C3, (**d**) specimen type C4.

The study of the critical state for numerical calculations made it possible to determine the geometric form of buckling and the corresponding critical load values for each stacking sequence of the composite material, as shown below (Figure 10 and Figure 11).

The study of the critical state of thin-walled B- and C-type columns showed high qualitative and quantitative convergence of the findings. The results of the numerical analyses made it possible to determine the forms of buckling and the corresponding critical load values. Therefore, the following results were determined for specimens with different fiber arrangements: specimen B1—three half-waves with critical load value *P*_cr_ = 20,359 N, specimen B2—four half-waves with critical load value *P*_cr_ = 19,556 N, specimen B3—five half-waves with critical load value *P*_cr_ = 22,336 N, and specimen B4—seven half-waves with critical load value *P*_cr_ = 17,753 N. 

Similar results were obtained for C-type columns. The values of the critical forces achieved and the number of half-waves are as follows for subsequent arrangements of composite layers: specimen C1—three half-waves with critical load value *P*_cr_ = 15,170 N, specimen C2—three half-waves with critical load value *P*_cr_ = 14,037 N, specimen C3—five half-waves with critical load value *P*_cr_ = 18,221 N, and specimen C4—six half-waves with critical load value *P*_cr_ = 13,937 N. It is worth noting that the number of half-waves obtained for layer arrangement 1 and 3 was the same; however, the loss of stability for type C columns occurred at a critical load 4–5 kN lower than for type B columns.

Qualitatively, the experimental tests and numerical simulations showed a high level of agreement. The high qualitative agreement between the results of numerical simulations and bench tests is shown in Table 4.

Based on the tests conducted, it was observed that the results of the numerical simulations slightly exceeded the value of the obtained forces in experimental tests. Higher values of critical loads in the case of simulations were due to the fact that in numerical simulations perfectly reflected physical models were considered but without geometric imperfections due to manufacturing technology. These models were characterized then by a slightly higher stiffness, which translated into the values of the obtained forces. A direct comparison of the results of the two types of analysis showed a discrepancy in the range of 2–6%. In the case of type C3 specimens, the critical load obtained in FEM simulations was 1.06 times higher than that obtained from the average result (of three specimens) from experimental tests. The remaining results had a much smaller error, indicating a high convergence of the obtained quantitative results. The highest value of critical load was observed for sample type B3: *P*_cr_ = 22,336 N—FEM, *P*_cr_ = 21,947 N—mean value EXP.

The findings presented in this paper were the result of research work carried out within the framework of a project financed with resources from the National Science Centre with registration number 2021/41/B/ST8/00148.

## 6. Conclusions

The research presented in this article constitutes a buckling analysis of thin-walled composite columns with rectangular cross-sections. The study of two types of columns (B and C) investigated four different layer arrangements (lay-ups). The analyses carried out involved physically manufactured structures as well as numerical simulations using the finite element method. The research was carried out using interdisciplinary testing techniques using a universal testing machine, an optical deformation measurement system, and numerical simulations using FEM. Evaluation of the achieved results was conducted qualitatively (percentage discrepancies) and quantitatively (several samples of profiles with the same layer stacking). The study showed that the highest stability is characterized by columns with an arrangement of layers defined by the number 3 [45°/−45°/90°/0°]s for both type B and C columns. It is worth noting that thin-walled structures with a shape closer to a square (type B) show higher values of the critical load at which buckling of the column occurs. Thin-walled structures of type B showed an average of 4–5 kN higher critical load value than type C columns. The specimens characterized by the lowest critical load values had a lay-up of [0°/90°/0°/90°]s for the type B column and [90°/−45°/45°/0°]s for the type C column. Noteworthy is that the type C column with a cross-section of 20 × 60 mm had similar critical load values for the C2 and C4 systems. All the results obtained through the numerical analyses as well as the bench tests are characterized by high quantitative and qualitative agreement. The presented results describe the critical condition of thin-walled composite columns, and this is the first stage of the work. The next stage of the work in the next article will realize the study of the load capacity of the structure using numerical simulations, taking into account the failure of composite materials such as CZM, XFEM, PFA, or LaRC05, among others [46,47,48,49].

## Figures and Tables

**Table 1:** Material properties of the carbon–epoxy composite—average values (with standard deviation).

Mechanical Parameters		Strength Parameters	
Young’s modulus *E*_1_ [MPa]	103,014.11 (2145.73)	Tensile Strength *F*_TU_ (0°) [MPa]	1277.41 (56.23)
Young’s modulus *E*_2_ [MPa]	7361.45 (307.97)	Compressive Strength *F*_CU_ (0°) [MPa]	572.44 (46.20)
Poisson’s ratio *v*_12_ [-]	0.37 (0.17)	Tensile Strength *F*_TU_ (90°) [MPa]	31.46 (9.64)
Kirchhoff modulus *G*_12_ [MPa]	4040.53 (167.35)	Compressive Strength *F*_CU_ (90°) [MPa]	104.04 (7.34)
-	-	Shear Strength *F*_SU_ (45°) [MPa]	134.48 (2.71)

**Table 2:** Critical state results for column type B—experimental studies.

	Specimen No.	1	2	3	Average Value
Specimen Type					
B1		19,932 N	19,716 N	19,837 N	19,829 N
B2		18,544 N	18,892 N	18,771 N	18,736 N
B3		21,654 N	22,054 N	22,133 N	21,947 N
B4		16,992 N	17,665 N	16,666 N	17,108 N

**Table 3:** Critical state results for column type C—experimental studies.

	Specimen No.	1	2	3	Average Value
Specimen Type					
C1		14,445 N	14,945 N	14,947 N	14,779 N
C2		13,818 N	13,352 N	13,284 N	13,485 N
C3		16,487 N	18,041 N	17,075 N	17,201 N
C4		13,864 N	13,656 N	13,091 N	13,537 N

**Table 4:** Critical state results—comparison of experimental studies and numerical simulations.

Specimen Type	Average Value *P*_cr_ (EXP) [N]	*P*_cr_ (FEM) [N]	FEM/EXP
B1	19,829	20,359	1.03
B2	18,736	19,556	1.04
B3	21,947	22,336	1.02
B4	17,108	17,753	1.04
C1	14,779	15,170	1.03
C2	13,485	14,037	1.04
C3	17,201	18,221	1.06
C4	13,537	13,937	1.03

## Data Availability

The data that support the findings of this study are available from the corresponding author, upon reasonable request.
